# Supplementary material for: Designing voice interfaces to support mindfulness-based pain management
Source: Digit Health. 2023 Oct 19;9:20552076231204418. doi: 10.1177/20552076231204418 (PMC10588404; doi:10.1177/20552076231204418)
Supplement: sj-docx-1-dhj-10.1177_20552076231204418 - Supplemental material for Designing voice interfaces to support mindfulness-based pain management [file sj-docx-1-dhj-10.1177_20552076231204418.docx]

Mindfulness Teacher Survey Survey Flow

**Standard: Consent (1 Question) Standard: Demographics (5 Questions) Standard: Pre-Interaction (3 Questions) Standard: Expert Testing (1 Question)**

**Standard: System Usability Scale (1 Question) Standard: Post-Interaction (3 Questions) Standard: Interview Request (1 Question) Standard: Contact Info (3 Questions)**

Page Break

**Start of Block: Consent**

This survey contains questions about your opinion on a new smart speaker skill for facilitating MBSR home practice. You will also be asked to interact with the skill on your Amazon Alexa device and share your thoughts about the prototype.

All of the responses you provide throughout the survey will be kept confidential, never be linked to you personally, and not be used for purposes other than this study. We will store your names and contacts in a database separate from the online questionnaire, so those who analyze it will not know who you are. Your participation in this study is voluntary. If you do not feel comfortable answering certain items or completing the survey in general, please notify the investigators.

The questionnaire should take about 30 minutes to complete in total. It is essential that you read the instructions for each section thoroughly before completing the questionnaire.

You can stop the survey at any time, and if you would like to share any concerns about the questionnaire after participating, please contact the investigator via e-mail.

Thank you very much for your cooperation and support of our work.

# Sanjana Mendu, [sanjana.mendu@psu.edu](mailto:sanjana.mendu@psu.edu) Saeed Abdullah, [saeed@psu.edu](mailto:saeed@psu.edu)

College of Information Sciences & Technology, Penn State University

# Sebrina Doyle Fosco, [sld40@psu.edu](mailto:sld40@psu.edu) Stephanie Lanza, [SLanza@psu.edu](mailto:SLanza@psu.edu)

Edna Bennett Pierce Prevention Research Center, Penn State University

*If you consent to participate in this research study, please click on the link "I agree".*

*Completion and return of the questionnaire will be considered your consent to participate in this study.*

- **I DO NOT AGREE** to participate in this study (1)
- **I AGREE** to participate in this study (2)

*Skip To: End of Survey If This survey contains questions about your opinion on a new smart speaker skill for facilitating M... = <strong>I DO NOT AGREE</strong> to participate in this study*

**End of Block: Consent**

**Start of Block: Demographics**

| 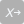 | 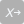 |
| --- | --- |

Sex What gender do you identify as?

- Male (0)
- Female (1)
- Non-binary (2)
- Prefer to self-describe (3)
- Prefer not to answer (-99)


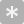


YOB What is your year of birth?

| 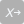 | 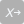 |
| --- | --- |

Ethnicity Which race/ethnicity do you identify with? [Check all that apply]

1. White (1)
2. Black or African American (2)
3. American Indian or Alaska Native (3)
4. Asian or Filipino (4)
5. Native Hawaiian or Other Pacific Islander (5)
6. Hispanic/Latinx (6)
7. Other (please describe): (7)
8. Prefer not to answer (8)


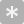


General Exp How many years have you been a mindfulness program facilitator?


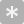


MBSR Exp How many years have you been a Mindfulness Based Stress Reduction (MBSR) program facilitator?

**End of Block: Demographics**

**Start of Block: Pre-Interaction**

Pre-Q1 What methods do you currently use for monitoring home practice and providing feedback while facilitating mindfulness programs?

Pre-Q2 What is your experience with smart speaker technology (e.g., Google Home, Amazon Alexa)?

Pre-Q3 What do you think of the role of smart speaker assistants in facilitating mindfulness practice?

**End of Block: Pre-Interaction**

**Start of Block: Prototype Testing**

**Instructions for Prototype Testing**

1. Log into your existing [Amazon Alexa account](http://alexa.amazon.com/) (or create a new one if you do not have one already)
2. [Click here to enable the skill on your Amazon Alexa account](https://skills-store.amazon.com/deeplink/tvt/389ce8d97f9f654eebda4efbc0a865111227590c0369c48df4b7455ba548af271169985b2978fca9f411b9122e62091dce81143f96f2eed8e8c04c42d6e8290f904b835aab2efef3fd0b46f19403d8614448c09e2f07e7ac806b3d10be8c0b9600b3aa55a6b4b190ae61fef95734f5b0)
3. Open the skill on your device by saying "Alexa, open mindful pain management"

Feel free to interact with the skill for as long as you want. Once you are ready to answer questions about your experience, please proceed to the next page.

**End of Block: Prototype Testing**

**Start of Block: System Usability Scale**

SUS

**System Usability Scale**

| Strongly disagree (9) | | Somewhat disagree (10) | Neither agree nor disagree (11) | Somewhat agree (12) | Strongly agree (13) |
| --- | --- | --- | --- | --- | --- |
| I think I would like to use the system frequently (344) | o | o | o | o | o |
| I found the system unnecessarily complex (345) | o | o | o | o | o |
| I though the system was easy to use (346) | o | o | o | o | o |
| I think that I would need the support of a technical person to use this system (347) | o | o | o | o | o |
| I found the various functions in this system were well integrated (348) | o | o | o | o | o |
| I thought that there was too much inconsistency in this system (349) | o | o | o | o | o |
| I imagine that most people would learn to use this system very quickly (350) | o | o | o | o | o |

| I found the system very awkward to use (351) | o | o | o | o | o |
| --- | --- | --- | --- | --- | --- |
| I felt very confident using the system (352) | o | o | o | o | o |
| I needed to learn a lot of things before I could get going with this system (353) | o | o | o | o | o |

**End of Block: System Usability Scale**

**Start of Block: Post-Interaction**

Post-Q1 Having used this technology, what do you think about using software like this to increase home practice?

Post-Q2 What impact do you think this could have for people going through MBSR?

Post-Q3 Based on your experience, what could be improved in this application?

**End of Block: Post-Interaction**

**Start of Block: Interview Request**

Interview Req Would you be willing to participate in a virtual interview?

- Yes (1)
- No (2)

*Skip To: End of Survey If Would you be willing to participate in a virtual interview? = No*

**End of Block: Interview Request**

**Start of Block: Contact Info**

Prompt Great! Please fill out the contact information form below, and a member of the study team will be in touch regarding next steps shortly


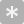


Name Name


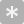


Email Email Address

**End of Block: Contact Info**
