# Supplementary material for: Designing voice interfaces to support mindfulness-based pain management
Source: Digit Health. 2023 Oct 19;9:20552076231204418. doi: 10.1177/20552076231204418 (PMC10588404; doi:10.1177/20552076231204418)
Supplement: sj-docx-2-dhj-10.1177_20552076231204418 - Supplemental material for Designing voice interfaces to support mindfulness-based pain management [file sj-docx-2-dhj-10.1177_20552076231204418.docx]

# INTERVIEW TOPIC GUIDE

Main questions formed the core structure of the interview,

further follow-up questions were put forward dependent on initial response.

# What methods do you currently use for monitoring home practice and providing feedback while facilitating mindfulness programs?

Example of follow-up questions:

- - *When do you employ these methods?*
  - *Does that provide the information that you need? Why?*

# What is your experience with smart speaker technology (e.g., Google Home, Amazon Alexa)?

Example of follow-up questions:

- - *If so, tell me what you know, how they’re used and your view of them.*
  - *Why do you have that view of them?*
  - *What do you think are the benefits / drawbacks of those you’re aware of?*

# What do you think of the role of smart speaker assistants in facilitating mindfulness practice?

Example of follow-up questions:

- - *You say you think it’d be useful, in what way could it help?*
  - *Why do you think smart speakers can play that role?*

------------------------- DEMONSTRATION OF EXEMPLAR SYSTEM PROVIDED -------------------------

# Having used this technology, what do you think about using software like this to increase home practice?

Example of follow-up questions:

- - Why does this system have that effect?
  - You think it’ll improve the quality of chronic pain management – How?

# What impact do you think this could have for people going through MBSR?

Example of follow-up questions:

- - *And what about any impact on your practice?*
  - *How do you see the difference using that might have on the way you work?*

# Based on your experience, what could be improved in this application?

Example of follow-up questions:

- - *What did you think about the way the information was presented?*
  - *Did you encounter any difficulties whilst using the software, any tasks that you found difficult or technical issues?*
  - *How would you comment on the user experience of the system?*
  - *What else needs to be considered in the design of the system?*
